# Pedigree with frontotemporal lobar degeneration – motor neuron disease and Tar DNA binding protein-43 positive neuropathology: genetic linkage to chromosome 9

**DOI:** 10.1186/1471-2377-8-32

**Published:** 2008-08-29

**Authors:** Agnes A Luty, John BJ Kwok, Elizabeth M Thompson, Peter Blumbergs, William S Brooks, Clement T Loy, Carol Dobson-Stone, Peter K Panegyres, Jane Hecker, Garth A Nicholson, Glenda M Halliday, Peter R Schofield

**Affiliations:** 1 Prince of Wales Medical Research Institute, Sydney, NSW, Australia; 2 University of New South Wales, Sydney, NSW, Australia; 3 Garvan Institute of Medical Research, Sydney, NSW, Australia; 4 SA Clinical Genetics Service, Women's and Children's Hospital, Adelaide, SA, Australia; 5 Institute of Medical and Veterinary Science, Adelaide, SA, Australia; 6 Neurosciences Unit, Department of Health, Perth, WA, Australia; 7 Neurodegenerative Disorders Research, Subiaco, WA, Australia; 8 College Grove Private Hospital, Adelaide, SA, Australia; 9 Northcott Neuroscience Laboratory, ANZAC Research Institute, Concord Hospital, Sydney, NSW, Australia; 10 Faculty of Medicine, University of Sydney, Sydney, Australia

## Abstract

**Background:**

Frontotemporal lobar degeneration (FTLD) represents a clinically, pathologically and genetically heterogenous neurodegenerative disorder, often complicated by neurological signs such as motor neuron-related limb weakness, spasticity and paralysis, parkinsonism and gait disturbances. Linkage to chromosome 9p had been reported for pedigrees with the neurodegenerative disorder, frontotemporal lobar degeneration (FTLD) and motor neuron disease (MND). The objective in this study is to identify the genetic locus in a multi-generational Australian family with FTLD-MND.

**Methods:**

Clinical review and standard neuropathological analysis of brain sections from affected pedigree members. Genome-wide scan using microsatellite markers and single nucleotide polymorphism fine mapping. Examination of candidate genes by direct DNA sequencing.

**Results:**

Neuropathological examination revealed cytoplasmic deposition of the TDP-43 protein in three affected individuals. Moreover, we identify a family member with clinical Alzheimer's disease, and FTLD-Ubiquitin neuropathology. Genetic linkage and haplotype analyses, defined a critical region between markers D9S169 and D9S1845 on chromosome 9p21. Screening of all candidate genes within this region did not reveal any novel genetic alterations that co-segregate with disease haplotype, suggesting that one individual carrying a meiotic recombination may represent a phenocopy. Re-analysis of linkage data using the new affection status revealed a maximal two-point LOD score of 3.24 and a multipoint LOD score of 3.41 at marker D9S1817. This provides the highest reported LOD scores from a single FTLD-MND pedigree.

**Conclusion:**

Our reported increase in the minimal disease region should inform other researchers that the chromosome 9 locus may be more telomeric than predicted by published recombination boundaries. Moreover, the existence of a family member with clinical Alzheimer's disease, and who shares the disease haplotype, highlights the possibility that late-onset AD patients in the other linked pedigrees may be mis-classified as sporadic dementia cases.

## Background

Frontotemporal lobar degeneration (FTLD) is the third most common neurodegenerative cause of dementia after Alzheimer's disease (AD) and dementia with Lewy bodies (DLB). [1,2] It stems from the degeneration of neurons in the superficial frontal cortex and anterior temporal lobes. Typically, this results in several distinct clinical presentations characterised by changes in personality and behaviour, including a decline in manners and social skills representative of frontotemporal dementia, as well as language disorders of expression and comprehension, known as progressive aphasia and semantic dementia, respectively. [3] Contributing to the spectrum of clinical phenotypes seen in FTLD is the co-occurrence of FTLD with motor neurone disease (MND). [4] MND, also referred to as amyotrophic lateral sclerosis (ALS) is characterised by degeneration of upper and lower motor neurons, leading to progressive muscle wasting, weakness and spasticity which ultimately results in profound global paralysis and death, usually due to respiratory failure.

FTLD is also a pathologically heterogeneous disorder and can be categorised into cases without detectable inclusions known as dementia lacking distinctive histopathology (DLDH), cases with tau-positive pathology known as tauopathies, and the most frequently recognised cases have ubiquitin-positive, tau-negative inclusions (FTLD-U). [5] The TAR DNA binding protein (TDP-43) is a nuclear protein implicated in exon splicing and transcription regulation, [6] and was recently identified as a major protein component of the ubiquitin-immunoreactive inclusions characteristic of sporadic and familial FTLD-U, with and without MND, as well as in sporadic cases of MND [7-9]. Recently, mutations in the TDP-43 (TARDBP) gene have recently been reported in familial and sporadic forms of MND. [10-14]

There is increasing evidence that FTLD and MND may represent two phenotypic variants resulting from a common underlying genetic cause. This is supported by both the presence of ubiquitin/TDP-43 pathology and also by genetic loci on chromosome 9 in families with FTLD and MND. Hosler et al. [15] identified a region on chromosome 9q21-22 from linkage data from 5 American FTLD-MND families. Subsequently, both Vance et al. [16] and Morita et al. [17] reported linkage to chromosome 9p13.2-21.3 in large FTLD-MND kindreds from Holland and Scandinavia, respectively. Finally, three other families were identified by Valdmanis et al. [18] with linkage to the chromosome 9p locus. Yan et al. [19] have also provided a preliminary abstract report of significant linkage in 15 FTLD-MND families. To date, only one gene, IFT74 has been postulated to be the causative gene of chromosome 9p-linked FTLD-MND. [20] However, only a single family has been identified with a mutation in the IFT74 gene, suggesting genetic heterogeneity in this region. Here, we report a large FTLD-MND family from Australia with linkage to chromosome 9p21.1-q21.3 and TDP-43 positive pathology, further supporting the evidence for a novel gene associated with this type of neurodegenerative disorder.

## Methods

### Neuropathology

The brains of patients III:2, III:3 and III:12 and the spinal cord of patient III:12 were obtained at the time of autopsy with consent. Routine neuropathological assessment, including immunohistochemical screening, was performed and reviewed and standardised for the present study. For all cases, retrospective review of standardised immunoperoxidase slides using antibodies for tau (MN1020, PIERCE, USA, diluted 1:10,000/cresyl violet), ubiquitin (Z0458, DAKO, Denmark, diluted1:200/cresyl violet), Aβ (gift from Professor Masters, University of Melbourne, dilution 1:200/cresyl violet), and a-synuclein (610787, Pharmigen, USA, diluted1:200/cresyl violet) were undertaken as previously described. [21] TDP-43 protein was visualised following microwave antigen retrieval (sections were boiled for 3 min in 0.2 M citrate buffer, pH 6.0) using commercially available antibody (BC001487, PTG, USA, diluted 1:500), peroxidase visualisation and counterstaining with 0.5% cresyl violet. To determine final diagnoses all cases were screened using current diagnostic criteria for AD, [22] dementia with Lewy bodies, [23] FTLD, [9] MND, [24] and other neurodegenerative syndromes including corticobasal degeneration, [25] progressive supranuclear palsy [26] and vascular dementia. [27]

### Genetic analyses

The study was approved by the University of New South Wales Human Research Ethics Committee and complies with the guidelines of the National Health and Medical Research Council and the Helsinki Declaration. After written informed consent was obtained, blood was collected from 16 family members (seven of whom are affected) and DNA extracted. A 10 cM genome-wide scan was performed with microsatellite markers (ABI Prism Linkage Mapping Set, version 2.5, MD-10). Parametric pair-wise and multipoint LOD scores were calculated using the MLINK and LINKMAP computer programs in the LINKAGE 5.2 package. Autosomal dominant inheritance was assumed with age dependent penetrance, a phenocopy rate of 0.005, a disease gene frequency of 0.001 and allele frequencies derived from a normal Australian population. [28] Seven liability classes were established based on pedigree data with 1% penetrance – age < 25 years, 8% – between 26 and 34 years, 22% – between 35 and 44 years, 46% – between 45 and 54 years, 71% – between 55 and 64 years, 91% – between 65 and 74 years, and 95% – age > 75 years. Individuals were assigned a liability class based on age-of-onset for affected cases and age at last consultation for asymptomatic cases. High-resolution fine mapping was performed using microsatellite markers with an average heterozygosity of 0.79 and spaced no further apart than 2 cM. Haplotypes were constructed using Merlin (Version 2.01), double checked manually, and displayed using HaploPainter V.029.5. [29] The haplotype of individual III:5 was inferred from her spouse and offspring.

### Mutation screen of candidate genes

Intronic polymerase chain reaction (PCR) primers were designed to amplify each non-coding and coding exon, as well as flanking intronic sequence of candidate genes using the ExonPrimer program accessed using the UCSC Genome Bioinformatics Site (primer sequences available on request). PCR products amplified from genomic templates were sequenced using Big Dye Chemistry (Applied Biosystems). Total RNAs were extracted from lymphoblastoid cell lines or frozen brain tissue for RT-PCR analysis. 1 μg of total RNA from each sample was reverse transcribed using the Superscript III First Strand Synthesis System (Invitrogen) and a oligodT primer (Invitrogen), followed by PCR amplification using overlapping primers designed to amplify the entire coding sequence of each candidate gene (primer sequences available on request). Each overlapping pair was designed to avoid exon/intron boundaries in order to detect splicing mutations. Each PCR fragment was analysed for abnormalities by size fractionation using agarose gel electrophoresis.

## Results

### Clinical and neuropathological examinations of affected members

**Figure 1:**
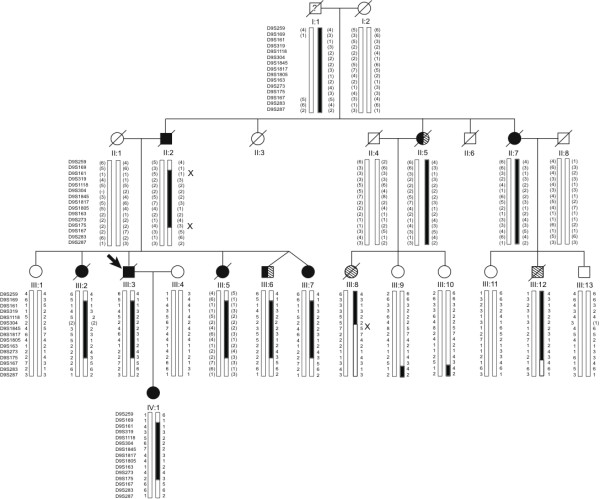
Pedigree diagram showing affection status and disease haplotype. Squares indicate males and circles females; filled arrow indicates proband; black symbols show individuals clinically diagnosed with dementia, either AD or FTLD; diagonal stripes, individuals diagnosed with MND; and combined black and diagonal stripes, individuals diagnosed with FTLD-MND. A diagonal line marks deceased subjects. Individual I:1, lived until his 80s, but was thought to have had some personality changes. Alleles in parentheses are inferred. **'X' **indicates upper and lower recombination breakpoints that define the minimal disease haplotype.

We describe an Australian family of Anglo-Celtic origin where eleven family members were affected with FTLD-MND (Figure 1). Over three generations, five family members (II:2, III:3, III:5, III:7, IV:1) presented with symptoms consistent with the behavioural variant of FTLD (Figure 1). Another two family members (III:8, III:12) presented with progressive bulbar and limb weakness consistent with MND. Two family members presented with a combination of FTLD and MND features (II:5, III:6). One of the other family member presented with early-onset dementia (II:7) and had a son with MND (III:12). Of particular interest is the eleventh affected family member. She presented with an amnestic picture and subsequently developed impairment in multiple cognitive domains including visuospatial function, prompting a clinical diagnosis of Alzheimer's disease (III:2). A full description of her clinical presentation is available [see Additional file 1]. Of the eleven affected family members, two also developed paranoid delusions in their middle age, at the beginning of their illnesses (III:6 and III:8). Average age of onset was 53 years (range 43 to 68 years) with a mean disease duration of 9 years (range 1 to 16 years), and mean age of death of 61 years (range 46 to 75 years). An overall clinical summary is provided in Table 1.

**Table 1:** Clinical summary

Individual	Gender	First symptoms	Disease duration	*APOE *genotype	Clinical presentation			
		(years)	(years)		Psychosis	FTD	ALS	Dementia*
II-2	M	~45	~18		-	+	-	-
III-3	M	~59	~7	e3/e4	-	+ ^#^	-	-
III-5	F	~45	~11		-	+	-	-
III-7	F	~59	2 (alive)	e3/e4	-	+	-	-
IV-1	F	43	3 (alive)	e3/e4	-	+	-	-
II-5	F	63	1		-	+	+	-
III-6	M	58	3 (alive)	e4/e4	+	+	+	-
III-8	F	46	4	e2/e4	+	-	+	-
III-12	M	46	5	e4/e4	-	-	+ ^#^	-
III-2	F	68	7	e3/e4	-	-	-	+ ^#^
II-7	F	50	16		-	-	-	+

* Refer to the text for details.

^# ^Diagnosis confirmed by neuropathological examination

**Figure 2:**
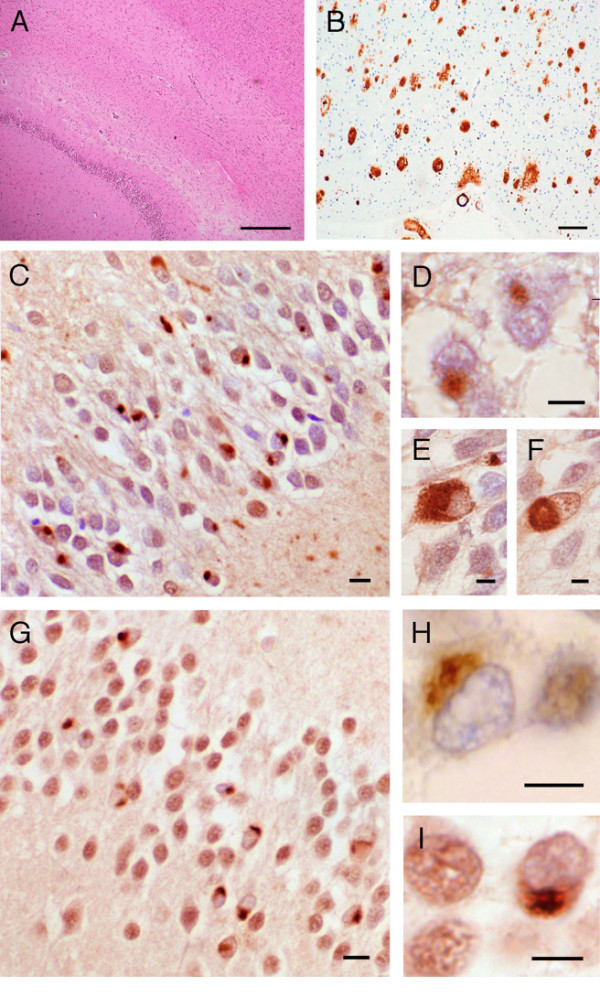
The neuropathology of patients III:2 and III:3. (**A**) Severe pyramidal neuronal loss from CA1 region Ammon's horn (III:2). (**B**) Temporal neocortex showing Aβ immunopositive plaques and cerebrovascular amyloidosis (III:2). Positive staining with ubiquitin (**C-F**) and TDP-43 (**G-I**) antibodies of neuronal cytoplasmic inclusions (NCI) in the granule cells of the dentate gyrus. Ubiquitin-positive neuronal cytoplasmic inclusions in III:2 (**C, E **and **F**) and in III:3 (**D**). TDP-43 positive neuronal cytoplasmic inclusions in III:2 (**G **and **I**) and III:3 (**H**). *Bar *= 50 μm in **A **and **B**; 20 μm in **C **and **G**; 10 μm in **D**, **E**, **F**, **H **and **I**.

**Figure 3:**
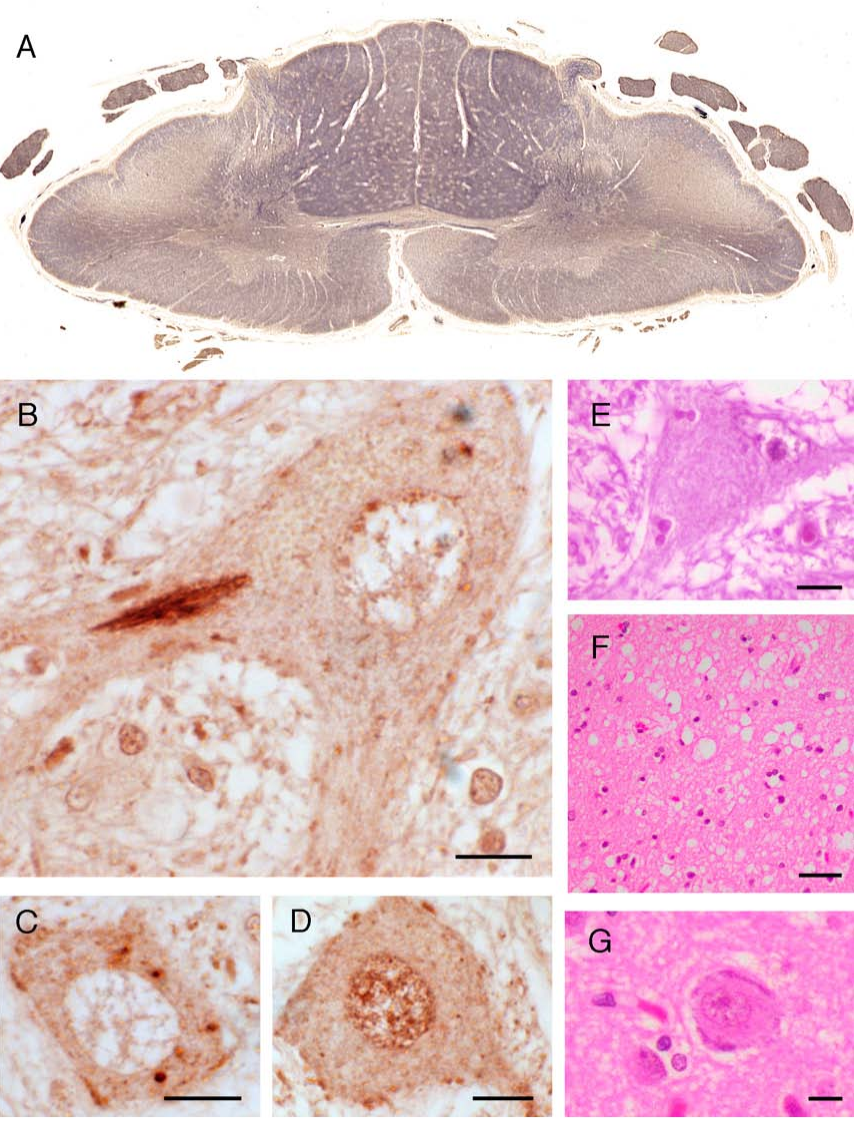
The neuropathology of case III:12. (**A**) C7 cervical cord showing symmetrical Wallerian degeneration of lateral corticospinal tracts and anterior corticospinal tract. Note atrophy of anterior nerve roots in comparison to dorsal nerve roots. TDP-43 immunopositive skein-like (**B**) and punctate (**C**) cytoplasmic inclusions within anterior horn cells of the spinal cord. (**D**) Normal TDP-43 positive nuclear staining of the anterior horn cell. (**E**) Anterior horn cell showing Bunina bodies. (**F**) Spongiosis in layers 2 and 3 of parasagittal motor cortex. (**G**) Residual Betz cell in motor cortex. *Bar *= 10 μm in **B**, **C**, **D**, **E **and **G**; 20 μm in **F**.

The location of the abnormal TDP-43-immunoreactive protein deposits within layer II neurons of the frontal cortex and hippocampal granule cells was identified as either cytoplasmic, intranuclear or neuritic. These features were used to classify the cases into histological subtypes according Cairns et al. [5] Histopathological examination was available for one family member with FTLD (III:3), finding TDP-43 inclusions consistent with type 2 FTLD-U [9] (Figure 2). Histopathological examination was available for one family member with MND (III:12), again finding TDP-43 inclusions in the dentate gyrus and anterior horn cells (Figure 3). The individual (III:2) with clinical Alzheimer's disease was found to have TDP-43 inclusions consistent with type 2 FTLD-U (Figure 2), with co-existing hippocampal sclerosis, as well as sufficient densities of cortical plaques and tangles but insufficient CA1 hippocampal neuritic pathology to fulfil criteria for Alzheimer's disease. Overall the severity of the FTLD-U histology for III:2 was more severe than the Alzheimer's disease histology. A detailed description of the clinical and pathological presentation of affected pedigree members is presented in Additional file 1.

### Linkage of causative locus to chromosome 9

DNA from the proband (III:3), III:6, III:12 and III:1 was subjected to DNA sequence analysis of the coding regions and flanking intronic sequences for known dementia and MND genes. No mutations were detected in the *APP, PSEN1, PSEN2, MAPT, PGRN, VCP, CHMP2B *or the *IFT74 *gene. No *SOD1 *or *TDP-43 *mutations were detected in individuals III:8 and III:12.

A genome-wide linkage analysis was undertaken on 16 pedigree members, some of whom are not included in the pedigree diagram for ethical reasons. Seven individuals were classed as affected and one was classified as unknown as she had psychosis, a possible FTLD prodromal feature. [16] Linkage analysis was carried out where a single genetic locus was considered causal for all clinical variants. Over the entire genome, the only region with a two-point LOD score greater than the established cut-off of 2.0 for suggestive linkage was located on chromosome 9. Marker D9S161 (9p21.3) gave a maximum LOD score of 2.57. Three adjacent markers also had positive LOD scores with the closest marker D9S1817 having a maximum LOD score of 0.99. The highest LOD score on a chromosome other than 9 was 1.40 on 3p14.3. Otherwise all other LOD scores were all consistently negative or non-significant and were used to exclude other reported MND linked loci, namely 2p13, 15q15-q22, 18q, 16q, and 20q13. These results indicate that the pedigree may be linked to the chromosome 9p FTLD-MND locus. The candidate chromosome 9p region was subjected to high resolution fine mapping with 8 additional markers (D9S259, D9S169, D9S319, D9S1118, D9S304, D9S1845, D9S1805, D9S163) surrounding D9S161 and D9S1817 and the data was re-analysed using MLINK. This resulted in a significant two-point LOD score of 3.25 at marker D9S319 (Table 2).

**Table 2:** Two-point LOD scores for chromosome 9p21.2-q22.32 markers

		III:8 Affected			III:8 Unaffected		
Marker	Location	θ			θ		
			
	(cM)	0.00	0.20	0.40	0.00	0.20	0.40

D9S259^‡^	47.19	-2.1	-0.07	0.03	-2.21	-0.09	0.03
D9S169^‡^	49.20	-0.54	0.53	0.08	-0.99	0.33	0.07
D9S161^†^	51.81	2.51	1.39	0.26	2.14	1.17	0.20
D9S319^‡^	54.50	3.25 *	1.96	0.51	2.82	1.70	0.43
D9S1118^‡^	58.26	1.08	0.59	0.13	1.05	0.57	0.43
D9S304^‡^	58.26	0.34	0.14	0.01	0.31	0.11	0.00
D9S1845^‡^	58.80	1.15	1.36	0.37	2.90	1.72	0.44
D9S1817^†^	59.34	1.47	1.55	0.43	3.24*	1.97	0.53
D9S1805^‡^	59.34	0.75	1.2	0.26	2.46	1.41	0.30
D9S163^‡^	59.87	1.34	0.53	0.04	0.94	0.31	0.00
D9S273^†^	65.79	1.03	1.36	0.37	2.77	1.62	0.40
D9S175^†^	70.33	0.50	1.01	0.28	2.26	1.40	0.36
D9S167^†^	83.41	-3.58	-0.30	0.01	-1.85	0.11	0.07

* Peak LOD Scores.

^† ^ABI Prism Linkage Mapping Set markers.

^‡ ^Marshfield Medical Research Foundation genetic framework markers.

All map distances are derived from the Marshfield Genetic Map except for marker D9S163 that was inferred from the Kong human genetic map. [33]

To further evaluate the reliability of the detected linkage, and to determine recombination breakpoints, haplotypes were constructed using Merlin (Figure 1). Recombination breakpoints were defined by two affected individuals. The telomeric boundary was marked by a recombination event in individual II:2 between markers D9S169 and D9S161, and has been inherited by all five affected offspring within the sibship (Figure 1). The centromeric boundary was defined by a single cross-over in individual III:8. However, using the microsatellite data, the exact recombination breakpoint could not be determined as markers D9S1118 and D9S304 are both homozygous for the '2' allele and could not be excluded from the disease haplotype. Therefore, we can only deduce with confidence that the cross-over occurred between markers D9S304 and D9S1845. All affected individuals share an identical haplotype consisting of 4 consecutive markers (D9S161-D9S319-D9S1118-D9S304) spanning a 9.6 cM region corresponding to a physical distance of 5.9 Mb.

### Fine mapping haplotype analysis and mutation screen of candidate genes

**Figure 4:**
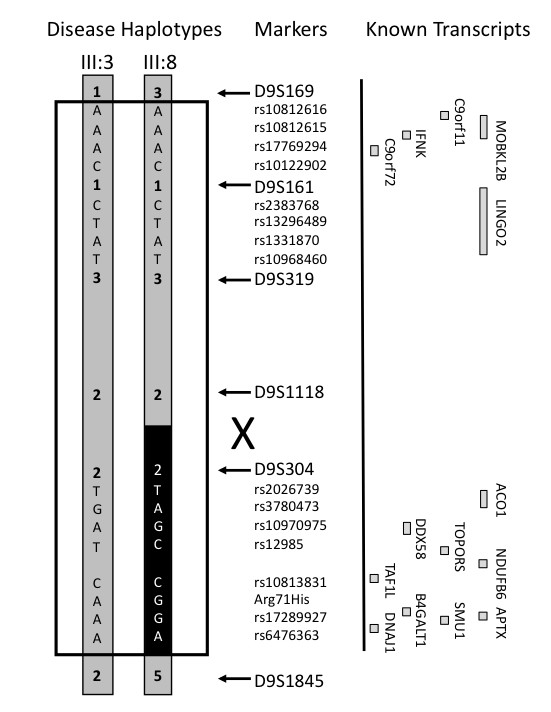
Fine mapping haplotype analysis using microsatellite and SNP markers to resolve the position of a meiotic recombination in pedigree member III:8. Four SNPs from representative genes are indicated (rs10812616, rs10812615, rs17769294 and rs10122902 for C9orf11; rs2383768, rs13296489, rs1331870 and rs10968460 for LINGO2; rs2026739, rs3780473, rs10970975 and rs12985 for ACO1; rs10813831, Arg71His, rs17289927 and rs6476363 for DDX8). The informative SNP haplotypes definitively place the recombination breakpoint between D9S1118 and D9S304. The black box indicates the portion of the disease haplotype which is not shared by pedigree member III:8. Transcript map indicating the relative positions of known genes and transcripts (open boxes) (not drawn to scale).

The 5.9Mb minimal disease region contains 14 known genes as listed by the UCSC Bioinformatics page, consisting of C9orf11 (ACR formation associated factor), *MOBKL2B, IFNK, c9orf72, LINGO2, ACO1, DDX58, TOPORS, NDUFB6, TAF1L, APTX, DNAJA1, SMU1*, and *B4GALT1 *(Figure 4). The coding and non-coding exonic sequence and flanking intronic regions of 11 of the initial set of candidate genes (excluding *TAF1L*, *SMU1 *and *B4GALT1*) were screened by direct sequencing of PCR products amplified from genomic template. Screening of the candidate genes detected 42 polymorphisms, of which six were considered novel, including two variants in C9orf11 (IVS1 +33 GT insertion/deletion, IVS4 -44 G/A); three in *DDX58 *(Arg71His CGT to CAT, IVS16 -23 C/A, IVS16 + 11 G/A) and one in *APTX *(IVS6 -12 insertion/deletion T). We considered the *DDX58 *amino acid change to be a polymorphism as it was found in unaffected and aged controls obtained from the Sydney Older Persons Study cohort [30] with a frequency of 0.03.

The additional SNP genotypes from the mutation screen were used to create an informative SNP haplotype and we were able to further fine map the centromeric recombination breakpoint. SNP haplotypes from *ACO1 *and *DDX58 *(the two genes in closest proximity to the D9S304 marker) revealed that III:8 did not inherit the same alleles of *ACO1 *and *DDX58 *as the other affected individuals. This allowed us to place the meiotic cross-over to between *ACO1 *and D9S304. As there are no known genes in the 60 kb region between *ACO1 *and D9S304, we have placed the final position of the cross-over to between D9S1118 and D9S304 (Figure 4). This haplotype analysis left 4 known genes (*IFNK*, *LINGO2*, *MOBKL2B*, C9orf11), and a hypothetical protein C9orf72 within the candidate region. No mutations were detected in the exons (coding and non-coding) or flanking intronic sequences of these five genes. In addition, we analysed three of the five genes/transcripts by RT-PCR of lymphoblastoid and brain cDNAs. The exceptions were C9orf11, which had been annotated to have testes specific expression (UCSC Bioinformatics Site), and *LINGO2*, whose coding sequence is encompassed within a single large 3' exon (UCSC Bioinformatics Site). No altered splicing or small-scale deletions in the RT-PCR products from the transcripts of these candidate genes were detected by size fractionation using agarose gel electrophoresis (data not shown). The absence of any mutations led us to conclude that III:8 may be a phenocopy and that the centromeric recombination breakpoint defined by that individual (between D9S1118 and D9S304) is not valid for defining the minimal disease region.

### Identification of an extended disease haplotype

**Figure 5:**
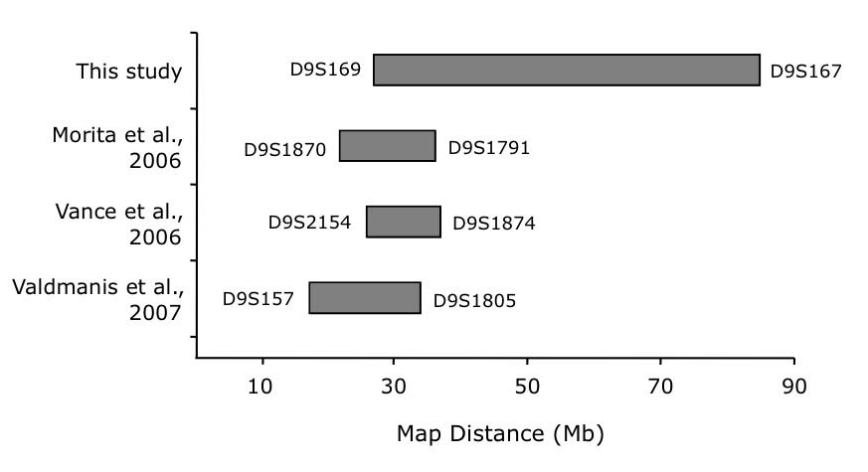
Diagram of chromosome 9p-linked families with FTLD-MND.

We re-analysed our linkage data with the phenotype of III:8 altered to an unaffected status using the same autosomal dominant inheritance model (Table 2). Again, only a single locus achieved a significant two-point LOD score of 3.24 at the marker D9S1817. The flanking markers, D9S1845 and D9S1805 also achieved positive LOD scores of 2.90 and 2.46, respectively (Table 2). A multi-point LOD score of 3.41 was observed at marker D9S1817. From the haplotype analysis (Figure 1), a new extended disease haplotype was defined using the distal telomeric meiotic cross-over between markers D9S175 and D9S167 (Figure 1) as identified in seven affected individuals II:2, III:2, III:3, III:5, III:6, III:7 and IV:1. The disease haplotype spans 57 Mb (34 cM) on chromosomal region 9p21-9q12. This region overlaps the three previously reported FTLD-MND regions on chromosome 9p (Figure 5). The recombination breakpoint observed in this study at D9S169 narrows the telomeric boundary of the combined published minimal disease region by 1.1 Mb.

## Conclusion

Frontotemporal lobar degeneration (FTLD) is a clinically, pathologically and genetically heterogeneous disorder. To date, at least 22 families with FTLD and/or MND have now been reported with genetic linkage to chromosome 9p [13-16] providing strong evidence that an additional FTLD gene exists. In this study we describe a large Australian FTLD-MND family that shows linkage to the chromosome 9p21.1-21.2 locus. With a significant two-point LOD score of 3.24 and a multi-point LOD score of 3.41, this is the only study that have provided statistically significant evidence for linkage from a single pedigree, the other pedigrees having two-point LOD scores of 2.41 [16], 2.81 [18] and 2.33 [17]. This means that we can rely on our haplotype analysis with greater statistical certainty. In addition, the family shows considerable clinical heterogeneity, compared to some other families that have been linked to the same locus.

Our genome-wide linkage analysis led to the identification of a genetic locus on chromosome 9p21.1-9q21.3. The resulting 57 Mb disease haplotype region overlaps with three other FTLD-MND loci identified by Morita et al., [17] Vance et al., [16] and Valdmanis et al. [18] (Figure 5) providing further evidence for this region as the disease locus. In combination, the four linkage studies collectively define a likely disease haplotype of 7.0 Mb between D9S169 and D9S1805 (Figure 5). We note that this haplotype does not overlap with the most recent preliminary abstract report of a 7.4 cM haplotype on the 9p region by Yan et al. [31] although, it does overlap with a region that was originally reported in abstract form by Yan et al. [19] Given that Yan's latest reported region is probably based on recombination events drawn from multiple families it is possible that one of the defining break points may be a false positive due to the low statistical power of individual pedigrees, [31] or that a disease haplotype boundary was defined by a phenocopy as observed in our pedigree. The region defined by D9S169 and D9S1118 (Figure 4) harbours the five transcripts that have been thoroughly screened in this study and by Momeni et al. [20] with no plausible mutations having been detected.

Changes in personality and behaviour, motor dysfunction as well as Ub/TDP-43 positive pathology represent the core clinical and neuropathological features characteristic of FTLD-MND families linked to 9p. In this study we describe an FTLD-MND family with additional clinical and pathological findings, not previously described in the chromosome 9p-linked families. Early and severe memory impairment is generally held to be an exclusion criterion for the clinical diagnosis of FTLD. [1] None of the other chromosome 9-linked pedigrees have reported major memory impairment as their primary diagnoses, although Morita et al. [17] mentioned that memory deficits were detected in three affected individuals in their pedigree. However this aspect was not reported as their primary diagnosis as their memory deficits were detected during neuropsychological tests three years before death. Moreover, Momeni et al. [20] reported that one of their patients had additional AD-like pathology, namely diffuse β-amyloid (Aβ) positive plaques in the absence of neuritic plaques and tangles. We too describe a patient (III:2) who presented with clinical symptoms typical of AD and at autopsy not only had indisputable TDP-43 positive neuronal cytoplasmic inclusions but also had amyloid-plaques and neurofibrillary tangles characteristic of AD (Figure 2). It has been postulated that Apolipoprotein E (APOE) may play a role in the development of Aβ deposition in FTLD cases [32]. There is no apparent association of APOE status with the presence of Aβ deposition in family 14 as the individual who was homozygous e4/e4 (III:12) had less Aβ deposition than the two individuals who were heterozygous e3/e4 (III:2 and III:3).

The first reported linkage of a novel FTLD-MND locus to chromosome 9p was in 2006, although no convincing candidate genes have yet been identified. The issue of phenocopies and the error of reliance on a single meiotic recombination events to define minimal disease regions could be a crucial factor in the failure to identify the disease gene. The recombination breakpoints reported in the literature by Morita *et al*. [17] and Valdmanis *et al*. [18] are based on a single recombination event in a single pedigree. Moreover, both of these pedigrees have two-point LOD scores less than 3. Vance et al. [16], using a pedigree with a LOD score of 2.4, showed recombination in multiple individuals. However, several of the individuals with the disease haplotype do not have FTLD-MND, [16] calling into question the relevance of this recombination breakpoint. Our reported increase in the minimal disease region should inform the other groups that the chromosome 9 locus may be more significantly more telomeric than predicted by the existing recombination breakpoints. Moreover, we report the existence of a case with clinical Alzheimer's disease, and FTLD-U neuropathology, who shares the disease haplotype. This result highlights the possibility that the classification of late-onset AD patients in the other linked pedigrees as sporadic dementia cases or unaffected may be erroneous, thereby reducing statistical power, or possibly even excluding pedigrees from linkage analysis. In summary, multiple families with FTLD-MND, without mutations in the known dementia genes, have been linked to chromosome 9p. This strongly suggests that the locus on chromosome 9 play a major role in pathogenic pathways that lead to FTLD-MND, making it imperative to identify the causative gene(s).

## Competing interests

The authors declare that they have no competing interests.

## Authors' contributions

JBJK, PRS conceived this study. AAL, CDS acquired the data. EMT, JH, GAN, WSB, PKP, CTL collected blood and clinical data from family. PB, GMH performed the neuropathological analyses. JBJK, AAL, PP, GMH and PRS participated in the mamangement, analysis, interpretation of data and drafting of manuscript. All have critically revised the manuscript for important intellectual content and seen and approved the final version.

## Pre-publication history

The pre-publication history for this paper can be accessed here:



## Supplementary Material

Additional file 1 Detailed clinical and neuropathological descriptions of pedigree members. Click here for file
