# Supplementary material for: Pedigree with frontotemporal lobar degeneration – motor neuron disease and Tar DNA binding protein-43 positive neuropathology: genetic linkage to chromosome 9
Source: BMC Neurol. 2008 Aug 29;8:32. doi: 10.1186/1471-2377-8-32 (PMC2553097; doi:10.1186/1471-2377-8-32)
Supplement: Additional file 1 — Detailed clinical and neuropathological descriptions of pedigree members. [file 1471-2377-8-32-S1.doc]

**Additional file 1**

*Detailed clinical and neuropathological descriptions of pedigree members*

**Case III:3 -** The proband (Fig. 1) presented at age 62 with definite and progressive behavioural changes for two and a half years, but the duration of illness was possibly longer. These changes were characterised by poor insight, loss of empathy, disinhibition (including sexual advances), deteriorating self-care and shop-lifting. Neuropsychological examination found impaired attention and working memory, marked deficits in frontal executive function with preservation of visuospatial and other posterior cortical functions. Taken together, his clinical findings were consistent with the behavioural variant of FTLD. He died of aspiration pneumonia aged 66**.**

At autopsy, the brain weighed 1243 grams and had no evidence of significant gyral atrophy. In particular the frontal lobes appeared macroscopically preserved. The ventricular system was symmetrical and of normal size. The basal ganglia, thalamus, hypothalamus and hippocampi were all normal. Histopathology was largely confined to cortical and medial temporal lobe structures with sparing of the basal ganglia, brainstem and cerebellum. There was ubiquitin- and TDP-43-immunopositive NCI in the entorhinal cortex, mild cortical vacuolation in the temporal pole, ‘granular’ NCI in the dentate granule cells (Fig. 2), granulovacuolar degeneration in the hippocampal CA1 pyramidal neurons, occasional ubiquitin- and TDP-43-immunopositive NCI in the superior frontal ßcortex, and mild spongiform change in the right parietal lobe. In the superior frontal and temporal neocortices, widespread diffuse plaque formation (up to 80 per X100 field) was observed following Aβ immunostaining. Rare tau immunopositivity was also present in the granular layer of the dentate gyrus, however no significant neurofibrillary tangle or Pick body formation was seen with either the Bielschowsky silver stain or immunostaining with AT8 (anti-phospho tau) antibody. No Lewy bodies were evident following α-synuclein immunostaining. The morphology and distribution of TDP-43 inclusions is consistent with type 2 FTLD-U histology according to the composite scheme adopted by Cairns *et al.* (2007).

**Case III:12 -** This man presented at age 45 with progressive lower limb weakness, necessitating a walking frame by age 47. By the age of 49 he had developed bulbar weakness, was unable to vocalise and required nasogastric tube feeding. Neurological examination found mixed upper and lower motor neuron signs including wasting, fasciculations and pathologically brisk reflexes in the tongue/jaw, upper and lower limbs. Verbal and written comprehension appeared intact, he was able to communicate by non-verbal means, and there were no unusual behaviours. His bedside findings satisfied the criteria for clinically definite MND. He died aged 51 of cardiorespiratory failure.

The brain weighed 1153 grams. External inspection of the left cerebral hemisphere showed mild atrophy of the left precentral gyrus. The left hemi-brain stem and cerebellum were normal. Coronal sections of the cerebral hemisphere were normal. Microscopic assessment revealed severe status spongiosis of layers 2 and 3 of the parasagittal motor cortex (Fig. 3) and mild spongiosis of middle and inferior portions of the motor cortex. There was subtotal loss of Betz cells (Fig. 3). There was mild patchy status spongiosis in layers 2 and 3 of the primary sensory cortex and mild focal spongiosis of the hippocampal CA1 neuropil. Many ubiquitin- and TDP-43-immunopositive, but α-internexin immunonegative NCI were present in the granule cells of the dentate gyrus. The Bielschowsky silver stains showed no plaques or tangles and AT8, Aβ and α-synuclein immunostains were negative. There was mild pallor of the mid-third of the cerebral peduncle (Weil stain) and of the medullary pyramidal tract consistent with descending Wallerian degeneration.

Macroscopic examination of the external spinal cord and segmental sections of the spinal cord showed atrophy of the anterior nerve roots. Microscopic examination of the spinal cord showed marked symmetrical loss of myelin staining (Weil stain) in the lateral and anterior corticospinal tracts (Fig. 3) consistent with descending Wallerian degeneration. There was severe loss of anterior horn cells which varied from segment to segment and side to side and was almost total in the cervical cord segments. Rare Bunina bodies were evident (Fig. 3) and skein-like cytoplasmic ubiquitin and TDP-43 immunopositive (Fig. 3) inclusions were present in some surviving anterior horn cells. There was marked atrophy of anterior nerve roots (Fig. 3). The neurons of the intermediolateral cell columns and Clarke’s column were intact.

**Case III:2 –** This woman's medical history included a dystonic neck tremor from age 35, which was also present in her mother and two of her five siblings (one with and one without FTLD-MND). She began repeating herself in conversation aged 68 and became increasingly forgetful after that. When assessed at the age of 69, she scored 18/30 on the mini-mental state examination. She was disoriented at the time and had difficulties with verbal recall, confrontational naming and visuospatial tasks. She started wandering a year later and was unable to self care by the age of 70. Her speech and verbal comprehension became limited by the time she was 72, and she was chairbound and no longer able to feed herself by the age of 74. When examined shortly before her death at age 75, she was mute and unable to obey simple verbal commands. There were myoclonic jerks involving her right upper and lower limbs, a dystonic neck tremor and she was hyper-reflexic in all four limbs with bilateral upgoing plantars. There were no fasciculations. She was diagnosed with myeloma soon after and died in a palliative care setting.

On examination, the brain weighed 936 grams. External inspection of the left cerebral hemisphere showed atrophy of the superior temporal gyrus and mild atrophy of the frontal lobe. The left hemi-brainstem and cerebellum appeared normal. A small hippocampus and amygdala was observed in coronal sections of the cerebral hemisphere. This corresponded with the microscopic finding of severe pyramidal cell loss from Ammon’s horn (Fig. 2), particularly involving the CA1 region, and the amygdala. No extracellular neurofibrillary tangles, TDP-43-, tau- or ubiquitin-immunoreactivity was evident in the region of hippocampal sclerosis. More abundant ubiquitin- and TDP-43-immunoreactive inclusions were found in the frontal and temporal sections sampled where there was severe loss of cortical neurons, prominent reactive fibrous astrogliosis and status spongiosus. These inclusions were similar in morphology to that seen in case III:3. However, the most significant depositions were found in the hippocampal dentate gyrus with a four-fold increase in ubiquitin/TDP-43-immunopositive NCIs (Fig. 2). A small number of these dentate NCI also showed moderate tau immunopositivity, although the greatest deposition of tau was observed in neuritic threads throughout the cerebral tissue sampled. Tau-positive neurofibrillary tangles were also found in some remaining hippocampal neurons, in the amygdala, and in neurons in most of the cortical regions sampled. There was an abundance of plaques and cerebrovascular amyloidosis, particularly in the temporal neocortex (Fig. 2). No significant abnormalities were observed in basal ganglia, brainstem or cerebellar regions, and no Lewy bodies were evident following a-synuclein immunostaining. The morphology and distribution of TDP-43 inclusions is consistent with type 2 FTLD-U histology according to the composite scheme adopted by Cairns *et al.* (2007). The case also had hippocampal sclerosis, as well as sufficient densities of cortical plaques and tangles to fulfil criteria for Alzheimer's disease (although not sufficient CA1 hippocampal neuritic pathology). Overall the severity of the FTLD-U histology was more severe than the Alzheimer's disease histology.

**Case III:6 -** This man first came to medical attention aged 58 with an acute psychotic episode characterised by auditory hallucinations and persecutory delusions involving his family and neighbours. With hindsight, this was preceded by subtle personality changes (poor empathy and overspending) for ten years, and impaired work performance with mild paranoia in the preceding twelve months. The psychotic episode resolved within a month and had not recurred despite his decision to stop risperidone of his own accord. In the two years following this he became increasingly impulsive, inflexible and uncaring. He overspent without discussion with his wife, undertook a number of unwise investment decisions and remained without insight. Neurological examination was normal until age 60 when frontal release signs were first noted. At the age of 61 he was found to have slurred speech with tongue fasciculations and wasting. Investigations at the time including lumbar puncture, routine Magnetic Resonance Imaging and an ^18^F-fluorodeoxyglucose Positron Emission Tomography scan were all within normal limits. Neuropsychological examination found borderline frontal executive function but impairment was not severe enough for a definitive diagnosis. In the ensuing twelve months, the patient developed mixed upper and lower motor neuron signs in both his upper and lower limbs. He is currently anarthric but still able to walk with a severe left foot drop.
